# Diagnostic Utility of the PD-L1 Immunostaining in Biopsy Specimens of Patients with Biliary Tract Neoplasms

**DOI:** 10.1007/s11605-021-05197-6

**Published:** 2022-02-08

**Authors:** Kazuyuki Matsumoto, Toshiaki Ohara, Masayoshi Fujisawa, Akinobu Takaki, Masahiro Takahara, Hironari Kato, Ryuichi Yoshida, Yuzo Umeda, Takahito Yagi, Akihiro Matsukawa, Hiroyuki Okada

**Affiliations:** 1 Department of Gastroenterology and Hepatology, Okayama University Graduate School of Medicine, Dentistry, and Pharmaceutical Sciences, Okayama, Japan; 2 Department of Pathology and Experimental Medicine, Okayama University Graduate School of Medicine, Dentistry, and Pharmaceutical Sciences, Okayama, Japan; 3 Department of Gastroenterological Surgery, Transplant and Surgical Oncology, Okayama University Graduate School of Medicine, Dentistry, and Pharmaceutical Sciences, Okayama, Japan

**Keywords:** Programmed death ligand 1, Bile tract neoplasm, Biopsy specimen, Immunohistochemistry

## Abstract

**Background:**

Anti-programmed death 1/programmed death ligand 1 (PD1/PD-L1) antibodies have been successfully used as treatment agents for several solid tumors; however, it is difficult to predict their effectiveness. We evaluated whether biopsy specimens could predict the positive status of PD-L1 in surgically resected tissue.

**Methods:**

Among 91 patients who underwent tissue sampling with endoscopic or liver biopsy before surgery for biliary tract neoplasms in an academic center, 45 (49%) patients were selected for retrospective analysis because the quality and quantity of their biopsy specimens were adequate for histologic evaluation. We performed immunohistochemical staining to investigate the PD-L1 expression in both resected and biopsy specimens. The percentage of the positively stained cells was calculated for subsequent use in the correlation investigation.

**Results:**

The biopsy methods were endoscopic retrograde cholangiopancreatography (ERCP) in 28 cases, percutaneous liver biopsy in 10 cases, and endoscopic ultrasound fine-needle aspiration in 7 cases. Among the 45 patients, when patients with > 10% positive tumor cells in surgically resected tissues were regarded as truly positive PD-L1, the positive and negative concordance rates between surgically resected tissues and biopsy samples were 56% (5/9) and 100% (36/36), respectively. With regard to the use of preoperative biopsy as a diagnostic tool, all (5/5) PD-L1-positive patients had a positive resected specimen. The accuracy of each biopsy method was as follows: ERCP, 89% (25/28); fine-needle aspiration, 86% (6/7); and liver biopsy, 100% (10/10).

**Conclusions:**

Biopsy samples could be a surrogate material for the assessment of the PD-L1 expression with substantial positive and high negative concordance rates.

**Supplementary Information:**

The online version contains supplementary material available at 10.1007/s11605-021-05197-6.

## Introduction

Intrahepatic cholangiocarcinoma (ICC), extrahepatic cholangiocarcinoma (ECC), and gallbladder carcinoma (GBC) are known biliary tract neoplasms arising from bile duct epithelial cells, collectively termed biliary tract cancer (BTC). ^1,2^ Neuroendocrine carcinomas (NECs) also rarely originate from the endocrine cells of the biliary tract, ^3^ whereas ampullary carcinomas (ACs) arise from epithelial cells in the papilla area. These tumors are pathologically diagnosed based on biopsy specimens obtained using endoscopic retrograde cholangiopancreatography (ERCP), endoscopic ultrasound fine-needle aspiration (EUS-FNA) or abdominal ultrasonography (US) guided transhepatic needle biopsy. Unfortunately, most of the cases are in their advanced stages due to the difficulty of early diagnosis. ^4^ While standard chemotherapies for patients with advanced BTC, including gemcitabine plus platinum, have been available, they have only been able to achieve an objective response rate (ORR) of 20%, a 5-year survival rate of < 10%, and a median overall survival (OS) time of 6–8 months. ^1,5–7^

Activated cancer-specific T cells significantly upregulate the expression of programmed death-1 (PD-1). PD-L1 is a ligand expressed on tumor cells and infiltrating immune cells to which PD-1 receptors bind. ^8–11^ Recently, therapeutic antibodies against PD1/PD-L1 have become available, to which approximately 20–30% of patients with several solid tumors, including bile duct neoplasms, have shown an effective tumor response. ^12,13^ In particular, patients with high-frequency microsatellite instability (MSI-H) and deficient DNA mismatch repair (MMR) have demonstrated notable response to immune checkpoint inhibitors (ICIs). ^14^ However, only 3.0% of those with solid tumors presented with MSI-H. ^15^

Extensive studies on PD-L1 immunohistochemistry (IHC) have also found it to be a viable predictive marker. ^16–19^ Previous results in patients with BTC have indicated that PD-L1 expression was associated with advanced stage and poor survival. ^20–23^ However, no study has established whether PD-L1 expression can be utilized as a biomarker for predicting the therapeutic effect of ICIs. ^24–26^ KEYNOTE-158 showed that PD-L1-expressers (*n* = 61) and PD-L1-nonexpressers (*n* = 34) had an ORR of 6.6% (4/61) and 2.9% (1/34), respectively, with no significant difference between both groups. ^24^ Moreover, KEYNOTE-028, ^25^ which exclusively enrolled PD-L1-positive patients, showed similar results to KEYNOTE-158, whereas another study showed that pembrolizumab provided lasting antitumor effect in 6–13% of patients with advanced BTC, regardless of PD-L1 expression. ^26^ However, all studies on PD-L1 expression had small sample sizes and involved a single arm. Thus, the predictive value, including PD-L1 cutoff points in BTC, requires further validation. Moreover, previous studies on PD-L1 expression by tumors and associated inflammatory cells utilized excised specimens and defined PD-L1 positivity as a combined positive score of ≥ 1%. ^24,25^ Given that the majority of biliary tract neoplasms are diagnosed at an advanced stage, evaluating PD-L1 expression without excision would certainly be beneficial for patients.

Therefore, the objective of the present study was to compare the PD-L1 expression in not only resected but also biopsy specimens from the same patients with biliary tract neoplasms in order to elucidate the relationship between PD-L1 expression and the patients’ clinicopathological features.

## Materials and Methods

### Patients

**Fig. 1:**
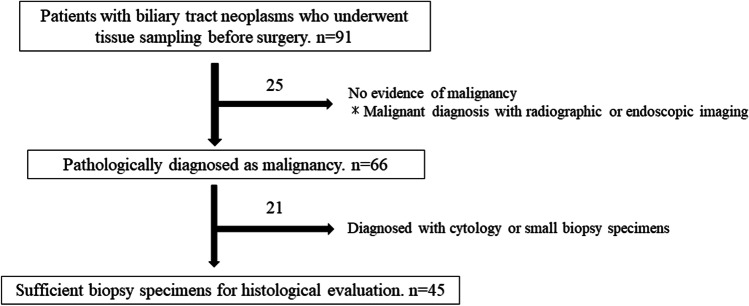
Study flow chart

A total of 91 patients with biliary tract neoplasms underwent tissue sampling with endoscopic or percutaneous liver biopsy before surgery at Okayama University Hospital between January 2012 and December 2018. The primary tumors were ECC, ICC, GBC, and AC in 33, 22, 22 (including 1 patient with NEC), and 14 patients, respectively. Among these patients, 45 (49%) with sufficient biopsy specimens for histological evaluation were retrospectively analyzed (Fig. 1). Biliary tract neoplasms were histologically confirmed using biopsy samples, after which all of the patients underwent upfront surgery without chemotherapy or radiotherapy. Resected cancers were classified in accordance with the International Union against Cancer, TNM Classification of Malignant Tumors, 8th edition. A prospectively maintained database was used for collecting data on the clinical parameters of all patients. OS was measured from the date the pathological diagnosis was established using the biopsy specimen until either the date of death or loss to follow-up. Informed consent was obtained from all patients before participation. Approval for the present study was obtained from the hospital’s Institutional Review Board for Human Research (IRB number, 2005–017) and was conducted following the guidelines of the Declaration of Helsinki.

### Tissue Biopsy Methods

#### ERCP

ERCP was performed by inserting a duodenal scope (TJF-260 V or JF-260 V; Olympus Medical Systems Corp.) into the papilla of Vater. After confirming the tumor-induced bile duct stricture, transpapillary biopsy was performed using forceps (Radial Jaw Biopsy Forceps 4P; cup diameter 1.8 mm, Boston Scientific Corporation, Japan) under radiological guidance following endoscopic sphincterotomy (EST). For tumors at the ampulla of Vater, direct biopsy using the same forceps with or without EST (EST for non-exposure type) was performed.

#### EUS-FNA

EUS was performed using a convex linear-array endoscope (GF-UCT260 or GF-UCT240; Olympus Medical Systems, Tokyo, Japan; EG-580UT; Fujifilm Corp., Tokyo, Japan) connected to a US device (EU-ME1 or EU-ME2 PREMIER PLUS; Olympus; SU-1 ultrasound processor; Fujifilm Corp.). A 22- and/or 25-gauge needle (EZ-Shot 3; Olympus Medical Systems Group, Tokyo, Japan; Acquire; Boston Scientific Corporation, Marlborough, MA, USA; Expect; Boston Scientific Corporation) was used for EUS-FNA. The white tissue parts obtained from the samples were used for histological evaluation, whereas the other parts were used for cytological evaluation. One cytology slide was subjected to modified Giemsa staining for rapid on-site evaluation and confirmation of the adequacy of the obtained sample.

##### Percutaneous Liver Biopsy

Percutaneous liver biopsy was performed using a 21-gauge needle (MAJIMA needle; Hakko Co., Ltd. Medical Device Division, Nagano, Japan) with a US device (Prosound SSD-α10; HITACHI Aloka, Tokyo, Japan). The biopsy technique using the MAJIMA needle was performed as follows: first, the fine needle was advanced into the target lesion. Second, the inner needle was pulled out, and negative pressure was produced with 30 mL of air. Third, the tumor was penetrated once. Fourth, the needle was turned once or twice and then pulled out from the liver. Finally, the tissue was pushed out using air.

For histological evaluation, tissues obtained using each biopsy method were promptly fixed in 10% paraformaldehyde and then embedded in paraffin for hematoxylin and eosin (HE) and IHC staining.

### Immunohistochemical Staining

All HE slides for each resected specimen were reviewed, after which one representative block was chosen for IHC staining. Anti-PD-L1 antibody was purchased from Cell Signaling Technology (catalog no. 13684; Danvers, MA, USA). Tissue sections were deparaffinized and blocked with 0.3% H_2_O_2_ in methanol at room temperature for 10 min. To retrieve antigen expression, the specimens were heated with a microwave in a Tris–EDTA buffer solution. Tissues were incubated with primary antibodies against PD-L1 at 4 ℃ for 24 h (1:200 dilution). Subsequently, a polymer detection system (Polink-2 Plus HRP detection kit) was used as per the manufacturer’s protocol (catalog no. D39-18; GBI Labs, Bothell, WA, USA).

### The Evaluation Protocol for IHC Findings

Two experienced pathologists blinded to the clinicopathological features of the patients were involved in independently determining the immunostaining signals. The proportion of the tumor and inflammatory cells (lymphocytes and macrophages) around the tumor site in each chosen field was determined by conducting a manual count of individual tumor cells using a × 10 objective lens. Tumor cells from the biliary tract cancers, especially well-differentiated ones, often had abundant intracytoplasmic mucin. As such, determining whether the signal was membranous or cytoplasmic was challenging in cases where the cytoplasm was occupied by mucin and cytosol was only present near the membrane. Only unequivocal membranous staining recognizable using a × 10 objective lens was regarded as a positive finding in this situation. Positive staining cutoff points was set at 5% and 10% based on previous reports concerning PD-L1 expression under immune checkpoint inhibitor usage. ^20,24,25,27–29^ As such, positive findings were herein defined as resected specimens with ≥ 5% or ≥ 10% PD-L1-positive tumors and inflammatory cells. Biopsy specimens were stained in a manner similar to resected specimens, after which the percentage of PD-L1 expression was determined. Determinations regarding the positivity of a staining were made based on a consensus between the two pathologists.

### Statistical Analyses

Continuous variables were expressed as median and interquartile range (IQR) or average and standard deviation (SD). Continuous variables were analyzed using Student’s *t* test, whereas categorical variables were analyzed using the chi-squared test. Cumulative survival rates were estimated using the Kaplan–Meier method. Significant differences in the survival status were assessed by Wilcoxon’s and the log-rank tests. All analyses were performed using the JMP Pro 15 software program (SAS Institute, Cary, NC, USA), with *P* values of < 0.05 indicating statistical significance.

## Results

### Patient Characteristics

The characteristics of the 45 patients are summarized in Table 1. Accordingly, the patients had a median age of 71 years (IQR, 66–77), with the following primary tumor distribution: ECC (*n* = 17), ICC (*n* = 11), GBC (*n* = 8), AC (*n* = 8), and NEC of the gallbladder (*n* = 1). PD-L1 IHC staining was successfully performed in all samples that could be histologically diagnosed as malignant, from which an average of 564 tumor and inflammatory cells were detected on biopsy specimens subjected to IHC. The rates of successful histological evaluation by IHC were 52% (17/33), 50% (11/22), 41% (9/22), and 57% (8/14) in patients with ECC, ICC, GBC, and AC, respectively.

**Table 1:** Characteristics of patients (*n* = 45)

Parameter	Number
Age, median (IQR), years	71 (66–77)
Sex, male, *n* (%)	21 (47)
Primary tumor, *n* (%)	
ECC	17 (38)
ICC	11 (24)
GBC^a^	9 (20)
AC	8 (18)
Biopsy site, *n* (%)	
Bile duct^b^	22 (49)
Liver	12 (26)
Ampulla of Vater	7 (16)
Lymph node	4 (9)
Size of biopsy lesion^c^, median (IQR), mm	30 (18–38)
Biopsy methods, *n* (%)	
ERCP	28 (62)
Percutaneous liver biopsy	10 (22)
FNA	7 (16)
Counted cells on biopsy average ± SD	564 ± 248
Tumor marker, median (IQR)	
CEA, ng/mL	2.7 (1.8–4.4)
CA19-9, U/mL	26 (11–115)
Pathological stage^d^	
I/II/III/IV	9 (20)/21 (47)/12 (27)/3 (6)
Lymph node metastasis positive, *n (%)*	20 (44)
Overall survival time, median (IQR), days	1121 (518–2551)

*ECC*, extrahepatic cholangiocarcinoma; *ICC*, intrahepatic cholangiocarcinoma; *GBC*, gallbladder carcinoma; *AC*, ampullary carcinoma; *IQR*, interquartile range; *ERCP*, endoscopic retrograde cholangiopancreatography; *FNA*, fine-needle aspiration; *SD*, standard deviation

^a^including one case with neuroendocrine carcinoma of gallbladder, ^b^including one case with biopsy at gallbladder

^c^measuring the stenosis length caused by carcinoma in cases with biopsy for bile duct

^d^UICC, International Union against Cancer, TNM Classification of Malignant Tumors, 8th edition

### PD-L1 Expression on Tumor Cells from Resected and Biopsy Specimens

**Fig. 2:**
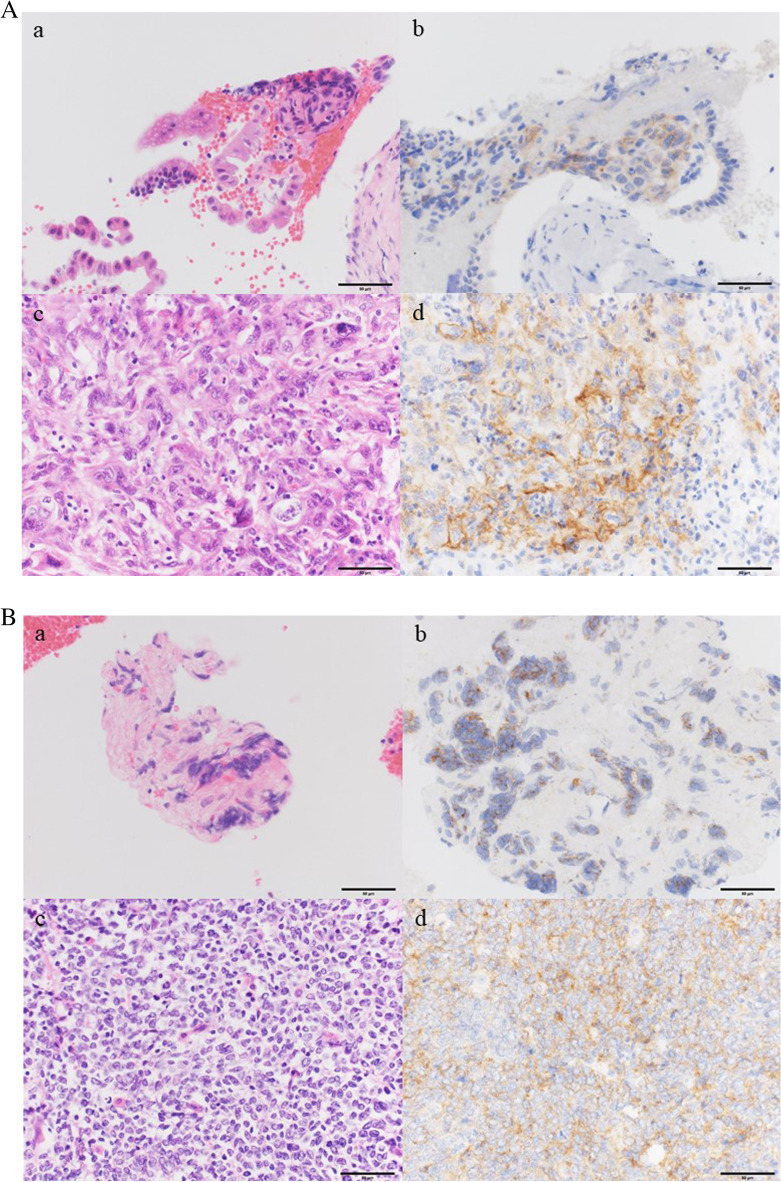

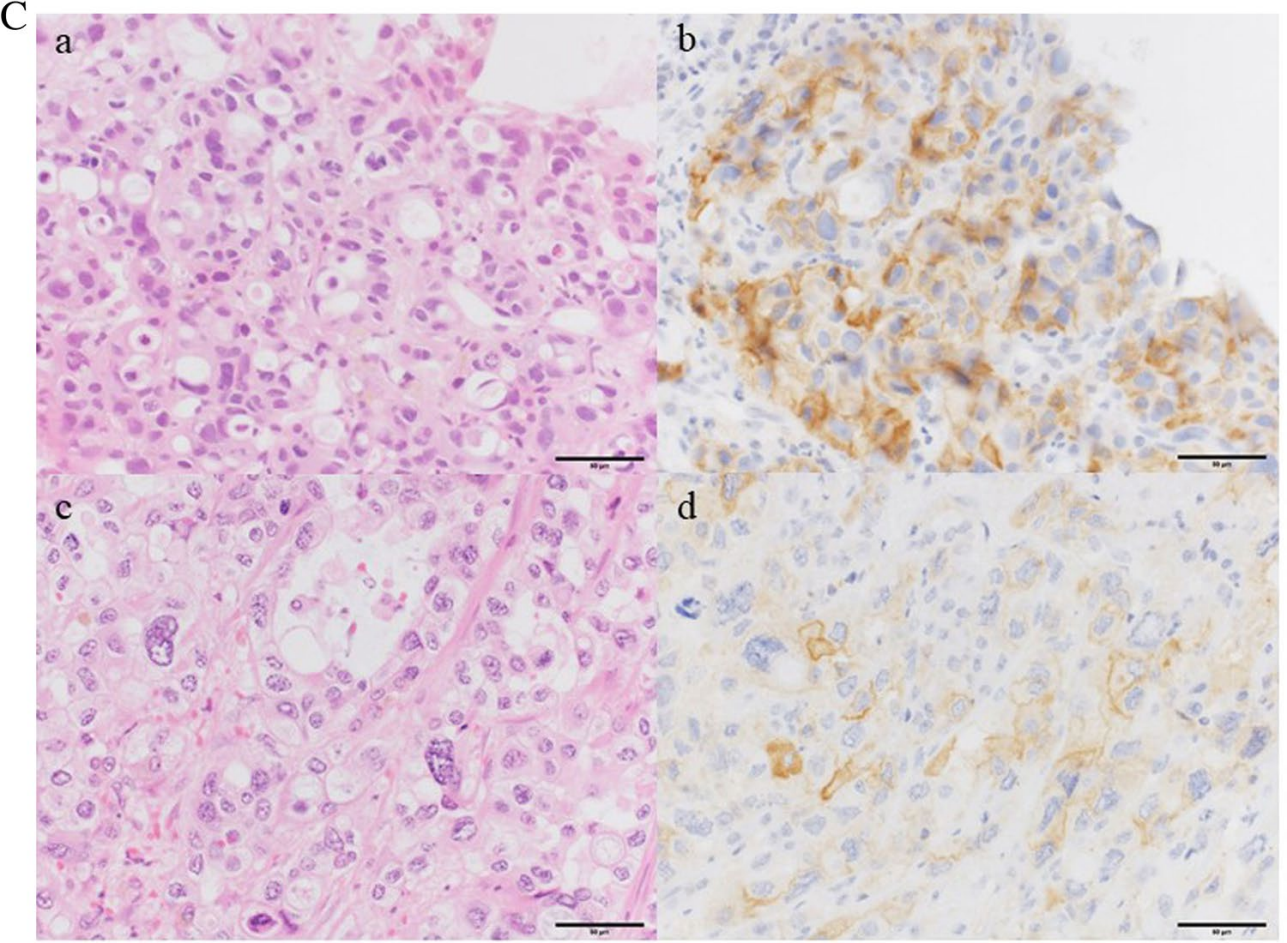
A Programmed death ligand 1 (PD-L1) staining with corresponding hematoxylin–eosin (HE) staining for extrahepatic bile duct carcinoma. PD-L1 and HE staining of a bile duct biopsy specimen obtained by endoscopic retrograde cholangiopancreatography (ERCP) (HE, **a**; PD-L1, **b**) and a resected specimen (HE, **c**; PD-L1, **d**). PD-L1 staining was primarily observed in the tumor cell membrane. Bars indicate 100 um. **B** PD-L1 staining with corresponding HE staining for neuroendocrine carcinoma. PD-L1 and HE staining of a lymph node specimen obtained by fine-needle aspiration (FNA) (HE, **a**; PD-L1, **b**) and a resected specimen (HE, **c**; PD-L1, **d**). PD-L1 staining was primarily observed in the tumor cell membrane. **C** Programmed death ligand 1 staining with corresponding HE staining for intrahepatic cholangiocarcinoma. PD-L1 and HE staining of a percutaneous liver biopsy (HE, **a**; PD-L1, **b**) and a resected specimen (HE, **c**; PD-L1, **d**). PD-L1 staining as primarily observed in the tumor cell membrane

Among the 45 patients, 11 (24%) and 9 (20%) were positive for PD-L1 expression on tumor cells from resected cancer specimens having cutoff values of 5% and 10%, respectively. After using cutoff values of ≥ 5% and ≥ 10%, the positive concordance rates for PD-L1 expression between resected and biopsy specimens were 46% (5/11) and 56% (5/9), whereas the negative concordance rates between the same were 94% (32/34) and 100% (36/36), respectively (Fig. 2). Using biopsy as a diagnostic tool, our results showed that all (5/5) patients whose biopsy specimens were positive for PD-L1 expression at a cutoff of ≥ 10% were also confirmed as positive using their resected specimens. The diagnostic accuracy of biopsy in the evaluation of PD-L1 expression on tumor cells in resected specimens is shown in Table 2 and Supplemental Table 1.

**Table 2:** The diagnostic accuracy of a biopsy for evaluating the PD-L1 expression on tumor cells in resected specimens (*n* = 45)

	No. of PD-L1 positive patients, (%)	Sensitivity, % (95% CI)	Specificity, % (95% CI)	PPV, % (95% CI)	NPV, % (95% CI)	Accuracy, % (95% CI)
Cutoff point ≥ 5% on resected and biopsy specimens	11 (24)	46 (25–58)	94 (88–98)	71 (39–91)	84 (78–88)	82 (72–88)
Cutoff point ≥ 10% on resected and biopsy specimens	9 (20)	56 (34–56)	100 (95–100)	100 (62–100)	90 (85–90)	91 (83–91)

*PPV*, positive predictive value; *NPV*, negative predictive value; *CI*, confidence interval; *PD-L1*, programmed death ligand 1

### PD-L1 Expression on Inflammatory Cells in Resected and Biopsy Specimens

**Fig. 3:**
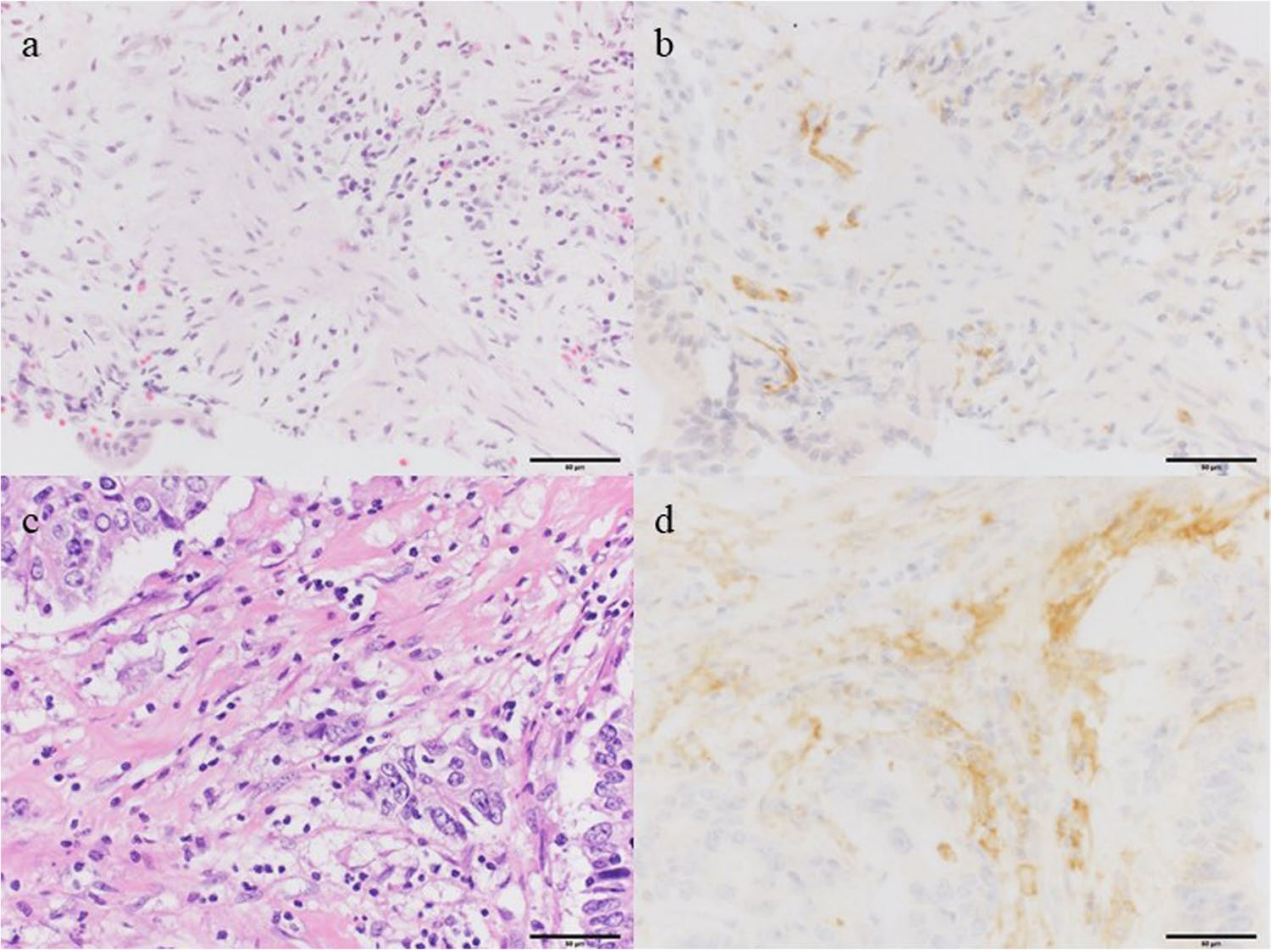
Programmed death ligand 1 (PD-L1) staining with corresponding hematoxylin–eosin (HE) staining for extrahepatic bile duct carcinoma. PD-L1 and HE staining of a bile duct specimen obtained by endoscopic retrograde cholangiopancreatography (ERCP) (HE, **a**; PD-L1, **b**) and a resected specimen (HE, **c**; PD-L1, **d**). PD-L1 staining was primarily observed in the inflammatory and stromal cell membrane. Bars indicate 100 um

Among the 45 patients, 28 (62%) and 23 (51%) had PD-L1-positive inflammatory cells in resected cancer specimens when cutoff values of 5% and 10% were used, respectively. On considering the patients truly positive for PD-L1 expression, the positive concordance rate for PD-L1 expression between the resected and biopsy specimens was 50% (14/28) and 52% (12/23), whereas the negative concordance rate between the same specimens was 71% (12/17) and 91% (20/22), respectively (Fig. 3). When using biopsy as a diagnostic tool, 86% (12/14) of the patients with PD-L1-positive biopsy specimen at a cutoff value of ≥ 10% were also confirmed as positive using their resected specimens. The diagnostic accuracy of biopsy in the evaluation of PD-L1 expression on inflammatory cells in resected specimens is shown in Table 3 and Supplemental Table 2.

**Table 3:** The diagnostic accuracy of a biopsy for evaluating the PD-L1 expression on inflammatory cells in resected specimens (*n* = 45)

	No. of PD-L1 positive patients, (%)	Sensitivity, % (95% CI)	Specificity, % (95% CI)	PPV, % (95% CI)	NPV, % (95% CI)	Accuracy, % (95% CI)
Cutoff point ≥ 5% on resected and biopsy specimens	28 (62)	50 (39–59)	71 (52–85)	74 (57–87)	46 (34–56)	58 (44–69)
Cutoff point ≥ 10% on resected and biopsy specimens	23 (51)	52 (39–58)	91 (78–97)	86 (65–96)	65 (55–69)	71 (58–77)

*PPV*, positive predictive value; *NPV*, negative predictive value; *CI*, confidence interval; *PD-L1*, programmed death ligand 1

### Accuracy of PD-L1 Expression on Tumor and Inflammatory Cells According to Biopsy Method and Site

The accuracy of PD-L1 expression on tumor and inflammatory cells (cutoff value, 10%) according to the biopsy method was as follows: ERCP, 89% (25/28) and 68% (19/28); FNA, 86% (6/7) and 43% (3/7); and liver biopsy, 100% (10/10) and 100% (10/10), respectively. Meanwhile, the accuracy of PD-L1 expression on tumor and inflammatory cells (cutoff value, 10%) according to the biopsy site was as follows: bile duct, 86% (19/22) and 73% (16/22); ampulla of Vater, 100% (7/7) and 57% (4/7); liver, 92% (11/12) and 92% (11/12); and lymph node, 100% (4/4) and 25% (1/4), respectively (Tables 4 and 5).

**Table 4:** Diagnostic accuracy of the PD-L1 expression on tumor and inflammatory cells by each of the biopsy methods

Positive cutoff point ≥ 10%		Biopsy methods		
		ERCP	FNA	Percutaneous liver biopsy
Accuracy	Tumor cells	89% (25/28)	86% (6/7)	100% (10/10)
	Inflammatory cells	68% (19/28)	43% (3/7)	100% (10/10)

*PD-L1*, programmed death ligand 1; *ERCP*, endoscopic retrograde cholangiopancreatography; *FNA*, fine-needle aspiration

**Table 5:** Diagnostic accuracy of the PD-L1 expression on tumor and inflammatory cells by each of the biopsy sites

Positive cutoff point ≥ 10%		Biopsy site			
		Bile duct	Ampulla of Vater	Liver	Lymph node
Accuracy	Tumor cells	86% (19/22)	100% (7/7)	92% (11/12)	100% (4/4)
	Inflammatory cells	73% (16/22)	57% (4/7)	92% (11/12)	25% (1/4)

*PD-L1*, programmed death ligand 1

### PD-L1 Expression and Clinicopathological Features

**Fig. 4:**
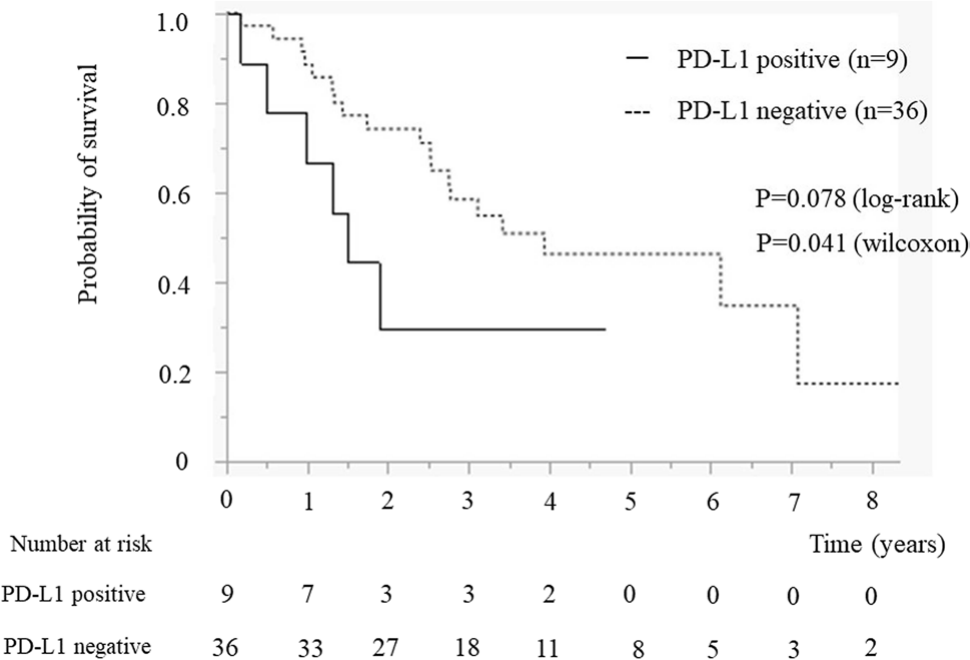
The overall survival time was evaluated by the Kaplan–Meier method. The median survival time was significantly shorter in the PD-L1-positive patients than in the PD-L1-negative patients (537 vs. 1418 days, *P* = 0.078, log-rank test, *P* = 0.041, Wilcoxon’s test)

The association between PD-L1 expression in the resected specimens and clinicopathological features is detailed in Table 6. Accordingly, PD-L1-positive patients had a significant larger tumor size compared to PD-L1-negative patients (36 vs. 21 mm, *P* = 0.035). PD-L1-positive patients showed a more advanced stage disease compared to PD-L1-negative patients (percentage of stage III–IV disease, 67% vs. 25%, *P* = 0.018). Survival analysis showed that PD-L1-positive patients had a significantly shorter median OS compared to PD-L1-negative patients (537 vs. 1418 days, *P* = 0.078, log-rank, *P* = 0.041, Wilcoxon’s test) (Fig. 4). Among patients with stage I–II disease, those who were PD-L1-positive tended to have a shorter median OS compared to those who were PD-L1-negative (537 vs. 2206 days, *P* = 0.13, log-rank, *P* = 0.083, Wilcoxon’s test). Among patients with stage III–IV disease, no significant difference was observed between those who were PD-L1-positive and PD-L1-negative (575 vs. 481 days, *P* = 0.99 by the log-rank test, *P* = 0.71 by Wilcoxon’s test) (Supplemental Fig. 1).

**Table 6:** Relationship between the PD-L1 expression and clinicopathological features

Variable	PD-L1 expression ( +) *(n* = 9)	PD-L1 expression ( −) (*n* = 36)	*P* value
Age, median (IQR) (years)	70 (57–75)	72 (67–77)	0.26
Sex, male/female	2/7	17/19	0.10
Tumor size, median (IQR), mm	36 (30–55)	21 (15–34)	0.035
Primary tumor			
ICC	2	9	0.14
ECC	3	14	
GBC*	4	5	
AC	0	8	
Tumor marker, median (IQR)			
CEA, ng/mL	1.8 (1.3–21)	2.7 (1.9–3.8)	0.68
CA19-9, U/mL	24 (18–195)	26 (11–65)	0.25
UICC classification			
I–II	3	27	0.018
III–IV	6	9	
Lymph node metastasis			
Positive	6	14	0.13
Negative	3	22	
Overall survival time, median (IQR), days	537	1418	0.041

*PD-L1*, programmed death ligand 1; *IQR*, interquartile range; *ICC*, intrahepatic cholangiocarcinoma; *ECC*, extrahepatic cholangiocarcinoma; *GBC*, gallbladder cancer; *AC*, ampullary cancer

PD-L1 expression ( +), PD-L1 was expressed ≥ 10% on resected specimen

^*^including one case having neuroendocrine carcinoma of the gallbladder

The subanalysis after excluding patients with AC revealed that tumor size of PD-L1-positive patients was significantly larger than that of PD-L1-negative patients (36 vs. 25 mm, *P* = 0.046). The rate of stage III–IV disease was higher among the PD-L1-positive patients compared to the PD-L1-negative patients (67% vs. 29%, *P* = 0.040). The survival analysis revealed that the PD-L1-positive patients had a significantly shorter median OS compared to the PD-L1-negative patients (537 vs. 1232 days, *P* = 0.095 by the log-rank test, *P* = 0.045 by Wilcoxon’s test) (Supplemental Fig. 2 and Supplemental Table 3). Among the patients with stage I–II disease, those who were PD-L1-positive tended to have a shorter median OS compared to those who were PD-L1-negative (537 vs. 2551 days, *P* = 0.14 by the log-rank, *P* = 0.062 by Wilcoxon’s test). Among the patients with stage III–IV disease, no significant difference in median OS was observed between those who were PD-L1-positive and those who were PD-L1-negative (575 vs. 648 days, *P* = 0.94 by the log-rank test, *P* = 0.69 by Wilcoxon’s test).

## Discussion

The current study has been the first to study PD-L1 expression concordance between surgically resected tissue and biopsy specimens from the same patients with biliary tract neoplasms. Deeming patients with > 10% positive tumor cells as truly positive for PD-L1, the current study found positive and negative concordance rates of 56% (5/9) and 100% (36/36), respectively. Using preoperative biopsy as a diagnostic tool, our findings showed that all (5/5) patients whose biopsy specimens were positive for PD-L1 expression also had a positive resected specimen. Therefore, the current study suggests the potential utility of biopsy in the area of precision medicine for patients with biliary tract neoplasms.

Matsumoto et al., who previously reported about the association between the PD-L1 expression of surgically resected specimens and FNA specimens in 94 patients with pancreatic cancer, ^30^ revealed positive and negative concordance rates of 44% (7/16) and 97% (76/78) at a cutoff of ≥ 5% and 55% (6/11) and 99% (82/83) at cutoff of ≥ 10%, respectively. Similar results using biopsy specimens had been found in pancreatic cancer. Considering the small size of the specimen, low sensitivity has remained a concern when using biopsy specimens. Given that biopsies obtain tumor cells from only a limited portion of the whole tumor, PD-L1-positive areas may not be detected unless such an area would be sampled. The expression of the resected specimens did not completely match the expression of PD-L1 assessed via IHC of biopsy specimens. In a previous study on pancreatic cancer, the patchy distribution of PD-L1 expression on tumors had been associated with false-negative results. ^30^ Notably, patients with false-negative results in the current study showed a similar patchy expression on the tumor, which was considered to be a risk factor for false-negative results. The current study also determined accuracy according to the biopsy method (i.e., ERCP, FNA, and percutaneous liver biopsy) and sites, with our findings showing relatively sufficient values across all methods and sites. In particular, specimens obtained via liver biopsy had been correctly diagnosed. This could have been attributed to the sufficient specimen size obtained for evaluation, which consequently reflected information for the whole tumor. On the other hand, biopsy specimens from the bile duct obtained via ERCP reflected only limited information regarding the luminal surface of the bile duct.

The concordance rate of inflammatory cells was insufficient, especially when using a low cutoff point for positive evaluation. According to biopsy site, the concordance rates of the bile duct, ampulla of Vater, and lymph node were also insufficient. Biopsy specimens from the bile duct and ampulla of Vater can be affected by the presence of bile and/or duodenal juice. Moreover, the inflammatory cells present in the swollen lymph node may be a product of not only the pure immunoreaction response to the tumor but also simple inflammation due to cholangitis. These differences may be associated with the low concordance rate of inflammatory cells between biopsy and resected specimens. Moreover, given that biopsy was basically performed at the tumor site, PD-L1 expression on tumor cells could be considered relatively accurate. PD-L1 expression on inflammatory cells reflects secondary changes around the tumor and may be difficult to evaluate using biopsy specimens obtained from the tumor site.

One study reported that the frequency of PD-L1 positivity (defined as ≥ 5% staining of tumor cells on IHC) in surgically resected specimens obtained from 652 patients with BTC was 8.6%, whereas that in GBC, ICC, and ECC was 12.3% (25/203), 7.3% (27/372), and 5.2% (4/77), respectively.^27^ Another study that analyzed 70 extrahepatic BTC specimens revealed that 43% were PD-L1-positive (defined as ≥ 3 + strongly positive immunohistochemical staining).^29^ Meanwhile, the current study found that 11 (24%) and 9 (20%) of the 45 patients with biliary tract neoplasms were positive for the expression of PD-L1 on tumor cells at a cutoff of 5% and 10% (10 (28%) and 8 (22%) when limited to the 36 patients with BTC, respectively). Based on the range of frequencies for immunohistochemical PD-L1 expression reported previously, the PD-L1-positive rate obtained in the current study cohort can be considered valid.

ERCP for bile duct biopsy is widely performed for biliary strictures; however, malignant biliary lesions are difficult to distinguish from benign lesions through non-surgical methods. De Bellis et al. ^31,32^ found that the sensitivity of transpapillary aspiration bile juice cytology was 27% in a cohort of 351 cases, whereas the sensitivity rates of brushing cytology and forceps biopsy were 42% among 837 cases and 56% among 502 cases, respectively. Other cytological sampling methods, such as fine-needle aspiration, were reported to show a sensitivity of 26–62%. Obtaining adequate tissue by biopsy of the bile duct is technically challenging and thus poses an issue with the IHC-based evaluation of biopsy specimens from patients with biliary tract neoplasms.

Studies have showed that PD-L1 was associated with advanced stage and poor prognosis in BTC. ^20–23^ Indeed, the tumor size was noted to be significantly larger in PD-L1-positive patients than in PD-L1-negative patients (36 vs. 21 mm, *P* = 0.035) in this study. Moreover, PD-L1-positive patients presented with more advance disease stages compared to PD-L1-negative patients (I–II vs. III–IV, *P* = 0.0089). Furthermore, our survival analysis found that PD-L1-positive patients had a significantly shorter median OS compared to PD-L1-negative patients (537 vs. 1418 days, *P* = 0.041, Wilcoxon test). Despite the relatively small number of patients with stage I–II disease, the current study showed that among patients with stage I–II disease, those who were PD-L1-positive tended to have a shorter median OS compared to those who were PD-L1-negative (537 vs. 2206 days, *P* = 0.083, Wilcoxon test). Some reports have shown correlation between PD-L1 expression in the resected BTC tissue and OS. ^20^ Given that tumors expressing PD-L1 can escape from natural T-cell activation against tumor cells, cancer growth among patients with PD-L1-positive tumors cannot be controlled through the tumor immune reaction. As such, the stage and size of the tumor would likely progress, promoting poor prognosis, even among those with early-stage cancer. The aforementioned findings might explain the correlation between PD-L1 and survival. When used as neoadjuvant or adjuvant chemotherapy in combination with other drugs, ICIs might be effective in patients with early-stage disease.

The present study has some limitations worth noting. First, this was a retrospective, single-center study with a small sample size. Second, genetic testing for evaluating deficient MMR and MSI, which can be used as predictive markers for ICIs, was not performed. As such, further prospective multicenter studies with larger sample sizes are needed to validate our findings.

## Conclusions

In conclusion, biopsy samples could be a surrogate material for the assessment of the PD-L1 expression with substantial positive and high negative concordance rates. In particular, when the PD-L1 expression was positive in the biopsy specimen, then the resected specimen was also confirmed to be positive.

## Supplementary Information

Below is the link to the electronic supplementary material.

Supplementary file1 (DOCX 29 kb)

Supplementary file2 (JPG 105 kb)

Supplementary file3 (JPG 140 kb)

Supplementary file4 (JPG 151 kb)

## Data Availability

The datasets used and/or analyzed during the current study are available from the corresponding author on reasonable request.
