# Supplementary material for: Diagnostic Utility of the PD-L1 Immunostaining in Biopsy Specimens of Patients with Biliary Tract Neoplasms
Source: J Gastrointest Surg. 2022 Feb 8;26(6):1213–23. doi: 10.1007/s11605-021-05197-6 (PMC9184404; doi:10.1007/s11605-021-05197-6)
Supplement: Supplementary file 1 — Supplementary file1 (DOCX 29 kb) [file 11605_2021_5197_MOESM1_ESM.docx]

**Supplemental tables**

**S-1a.** Concordance of the PD-L1 expression on tumor cells between the resected and biopsy specimens

|  | | Resected specimen  (positive: ≥5%) | |
| --- | --- | --- | --- |
|  |  |  |  |
|  | | (+) | (−) |
| Biopsy specimen  (positive: ≥5%) | (+) | 5 | 2 |
|  | (−) | 6 | 32 |
| PD-L1: programmed death ligand 1 | | | |

**S-1b.** Concordance of the PD-L1 expression on tumor cells between the resected and biopsy specimens

|  | | Resected specimen  (positive: ≥10%) | |
| --- | --- | --- | --- |
|  |  |  |  |
|  | | (+) | (−) |
| Biopsy specimen  (positive: ≥10%) | (+) | 5 | 0 |
|  | (−) | 4 | 36 |
| PD-L1: programmed death ligand 1 | | | |

**S-2a.** Concordance of the PD-L1 expression on inflammatory cells between the resected and biopsy specimens

|  | | Resected specimen  (positive: ≥5%) | |
| --- | --- | --- | --- |
|  |  |  |  |
|  | | (+) | (−) |
| Biopsy specimen  (positive: ≥5%) | (+) | 14 | 5 |
|  | (−) | 14 | 12 |
| PD-L1: programmed death ligand 1 | | | |

**S-2b.** Concordance of the PD-L1 expression on inflammatory cells between the resected and biopsy specimens

|  | | Resected specimen  (positive: ≥10%) | |
| --- | --- | --- | --- |
|  |  |  |  |
|  | | (+) | (−) |
| Biopsy specimen  (positive: ≥10%) | (+) | 12 | 2 |
|  | (−) | 11 | 20 |
| PD-L1: programmed death ligand 1 | | | |

**S-3.** Relationship between PD-L1 expression and clinicopathological features in patients with BTC

| Variable | PD-L1 expression (+) *(n* = 9) | PD-L1 expression (−) (*n* = 28) | *P* value |
| --- | --- | --- | --- |
| Age, median (IQR) (years) | 70 (57–75) | 72 (68–77) | 0.24 |
| Sex, male/female | 2/7 | 15/13 | 0.10 |
| Tumor size, median (IQR), mm | 36 (30–55) | 25 (18–33) | 0.046 |
| Primary tumor |  |  |  |
| ICC | 2 | 9 | 0.27 |
| ECC | 3 | 14 |  |
| GBC* | 4 | 5 |  |
| Tumor marker, median (IQR) |  |  |  |
| CEA, ng/mL | 1.8 (1.3–21) | 2.7 (1.9–5.1) | 0.56 |
| CA19-9, U/mL | 24 (18–195) | 40 (12–82) | 0.43 |
| UICC classification |  |  |  |
| I–II | 3 | 20 | 0.040 |
| III–IV | 6 | 8 |  |
| Lymph node metastasis |  |  |  |
| Positive | 6 | 13 | 0.29 |
| Negative | 3 | 15 |  |
| Overall survival time, median (IQR), days | 537 | 1232 | 0.045 |
| PD-L1: programmed death ligand 1, BTC: biliary tract carcinoma, IQR: interquartile range, ICC: intrahepatic cholangiocarcinoma, ECC: extrahepatic cholangiocarcinoma, GBC: gallbladder cancer, PD-L1 expression (+): PD-L1 is expressed in ≥10% of the resected specimen  * Including one case having neuroendocrine carcinoma of the gallbladder | | | |
|  |  |  |  |

**Supplemental figure legends**

**S-1A:** The overall survival time for stage I–II patients was evaluated using the Kaplan–Meier method. The median survival time tended to be shorter in the PD-L1-positive patients than in the PD-L1-negative patients (537 vs. 2206 days, *P* = 0.13, log-rank test, *P* = 0.083, Wilcoxon’s test).

**S-1B**: The overall survival time for stage III–IV patients was evaluated using the Kaplan–Meier method. No significant difference was observed between the PD-L1-positive and PD-L1-negative patients (575 vs. 481 days, *P* = 0.99 by the log-rank test, *P* = 0.71 by Wilcoxon’s test).

**S-2:** The overall survival time in patients with BTC evaluated by the Kaplan–Meier method. The median survival time was significantly shorter in the PD-L1-positive patients than in the PD-L1-negative patients (537 vs. 1232 days, *P* = 0.095, log-rank test, *P* = 0.045, Wilcoxon’s test).
