# Supplementary figures and images for: Diagnostic Utility of the PD-L1 Immunostaining in Biopsy Specimens of Patients with Biliary Tract Neoplasms
Source: J Gastrointest Surg. 2022 Feb 8;26(6):1213–23. doi: 10.1007/s11605-021-05197-6 (PMC9184404; doi:10.1007/s11605-021-05197-6)

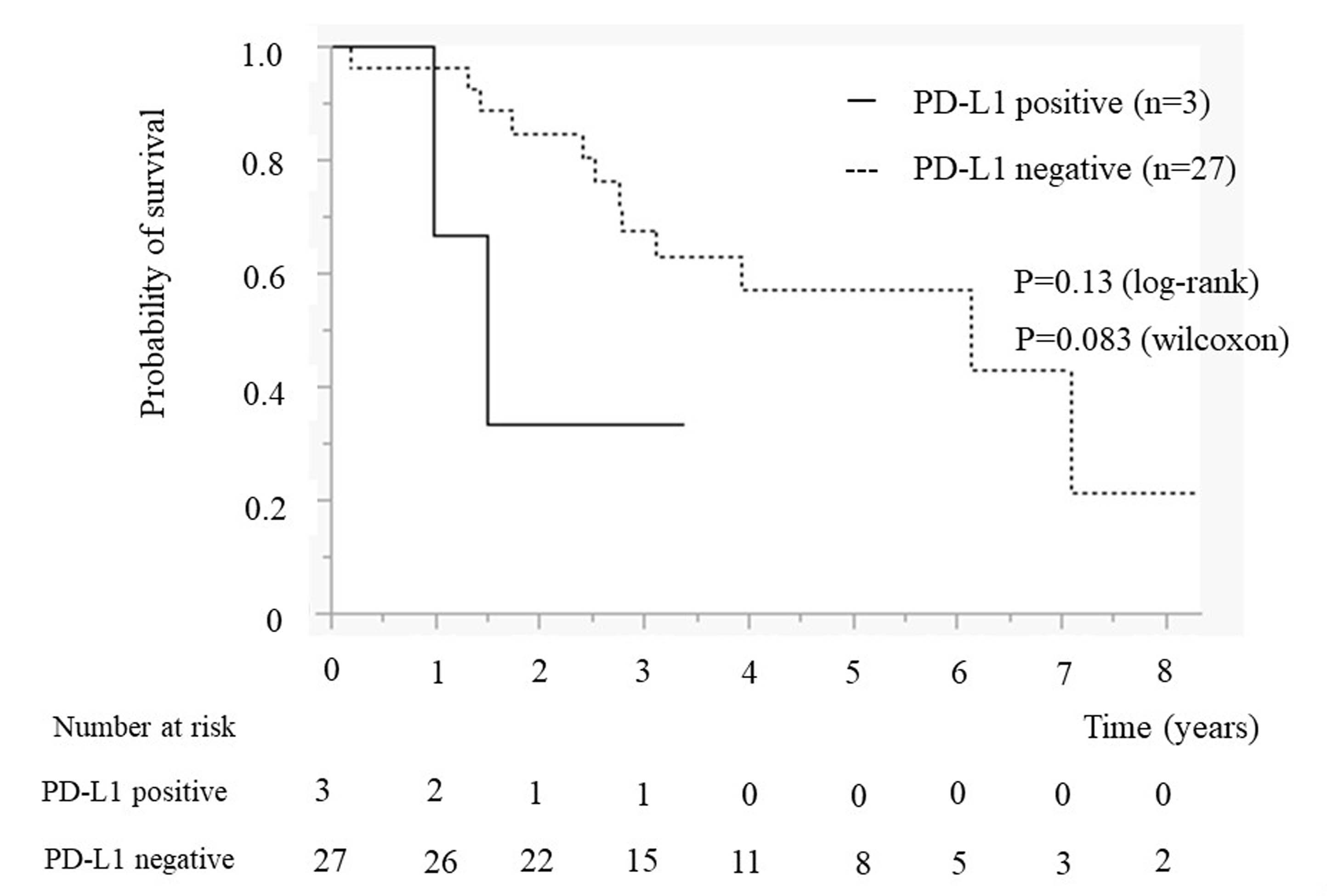

Supplement: Supplementary file 2 — Supplementary file2 (JPG 105 kb) [file 11605_2021_5197_MOESM2_ESM.jpg]

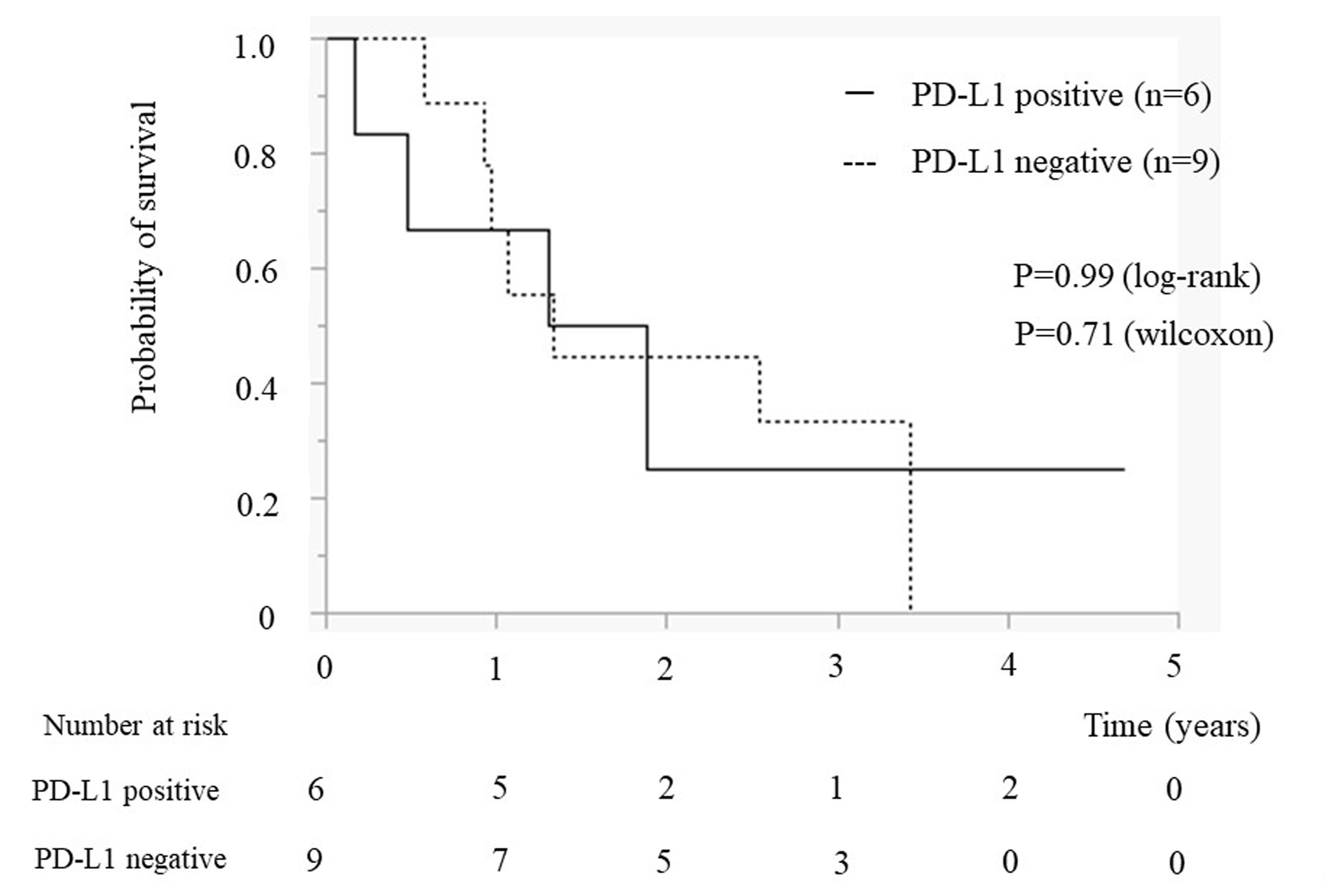

Supplement: Supplementary file 3 — Supplementary file3 (JPG 140 kb) [file 11605_2021_5197_MOESM3_ESM.jpg]

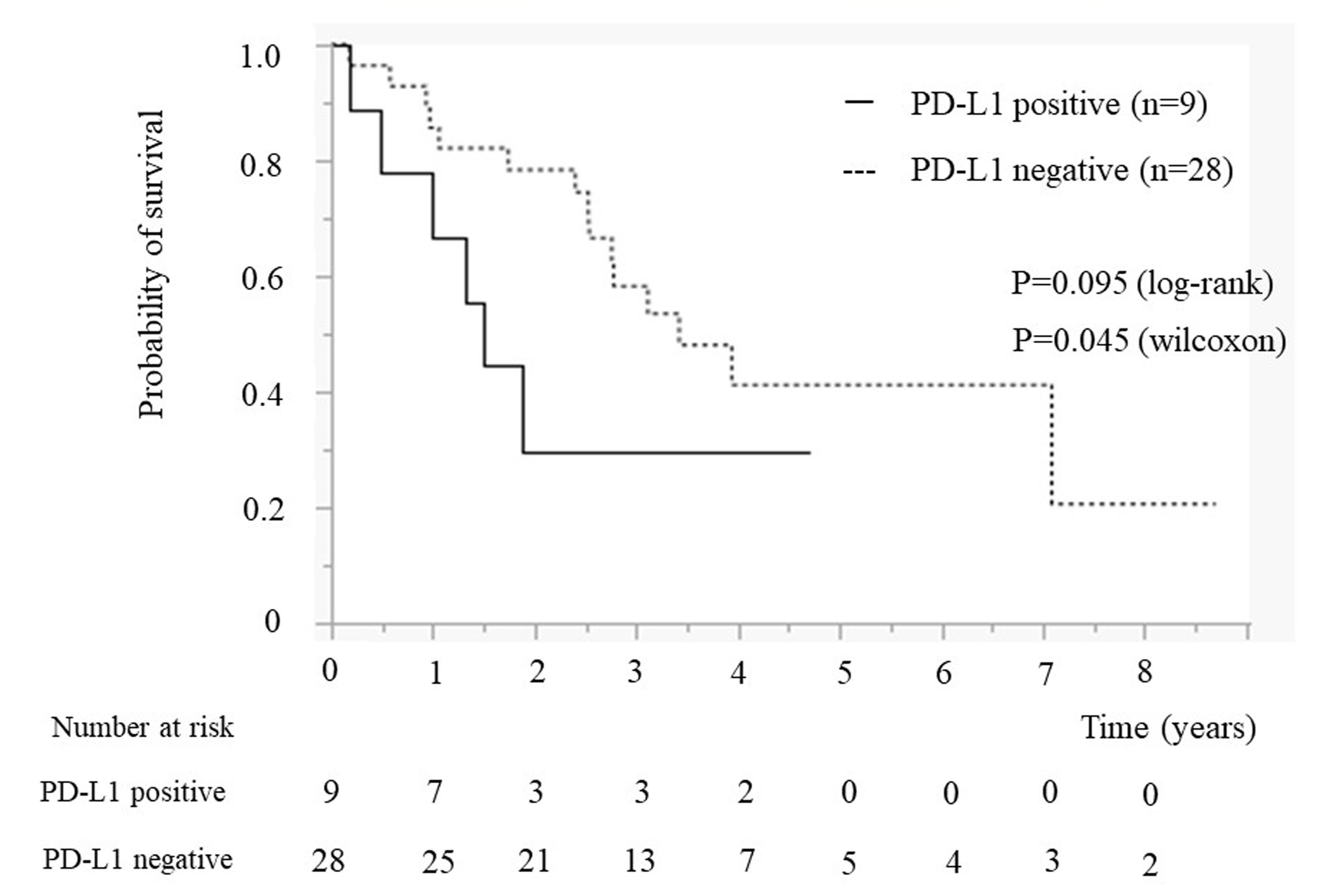

Supplement: Supplementary file 4 — Supplementary file4 (JPG 151 kb) [file 11605_2021_5197_MOESM4_ESM.jpg]
